# Community‐centred oral healthcare for adults experiencing homelessness in Australia: Perceptions and experiences of key stakeholders

**DOI:** 10.1111/hsc.14070

**Published:** 2022-10-14

**Authors:** Angela Durey, Helen Lette, Julie Saunders, Linda Slack‐Smith

**Affiliations:** ^1^ School of Population and Global Health The University of Western Australia Perth Western Australia Australia

**Keywords:** community care, homelessness, inequity, oral health, qualitative research

## Abstract

The objective of the study was to identify whether creating a responsive, respectful and trustworthy environment that provides free dental care for clients who are homeless using volunteer dental professionals was effective in meeting their oral health needs in Fremantle, Western Australia. Qualitative research conducted between October 2018 and August 2019 was guided by a social constructivist paradigm to gather and analyse data. Semi‐structured interviews were conducted with adults experiencing homelessness accessing a community dental clinic and health providers and other stakeholders involved in its establishment, management and service delivery. An inductive approach to analysis was used to organise themes under the categories of ‘establishing the oral health clinic’ (OHC) and ‘responses to the implementation of the clinic’ Thirty‐nine semi‐structured interviews were conducted across eight participant groups: clients, executive management, the oral health clinical reference group, volunteer dentists, employed staff, nursing students, volunteer staff and other stakeholders. Key findings across all groups included positive responses to the establishment and implementation of the OHC, the quality of care and the safe and respectful environment in which services were delivered. Challenges related to sustainability include uncertainty around ongoing funding and recruitment of dental professionals. Whilst volunteer dental services fill a gap in meeting the complex needs of this population group, mainstream services must consider and address issues of equity in this context. Findings can be used to guide this process that includes creating environments of respect and trust where adults who are homeless or at risk of homelessness feel safe, welcomed and more likely to return to the service.

**What is known about this topic**

- Oral health is poorer in adults who are homeless.
- Costs of dental treatment are often prohibitive for people experiencing homelessness.
- Adults who are experience homelessness often find it challenging to navigate dental services.

**What this paper adds**

- For adults who experience homelessness, this paper describes a community‐centred model of free dental care involving volunteer dentists.
- Findings indicated clients did not feel judged by health providers in this context
- Findings indicated a community‐based service could often better meet the needs of people experiencing homeless than centralised services.

## 1 INTRODUCTION

Homelessness is a health and social problem in Australia variously defined as sleeping rough outside or in improvised dwellings such as tents, overcrowded housing or temporary lodging (Australian Bureau of Statistics, 2018; Davies & Wood, 2018). Homelessness from living in overcrowded dwellings is increasing in Australia, particularly amongst adults aged 55–74 years (Australian Bureau of Statistics, 2018; Pawson et al., 2018). Growing numbers of adults are also at risk of homelessness due to family and domestic violence and structural issues such as poor housing affordability and precarious employment (Pawson et al., 2018). The effect of homelessness on a person's health and well‐being is described as ‘profound and compounding’ (Davies & Wood, 2018, p. 230). Adults experiencing homelessness are at greater risk of premature mortality, infectious diseases, mental health disorders, substance misuse and non‐communicable diseases (Fazel et al., 2014).

Oral health for adults facing homelessness may not be a priority with treatment often only accessed for pain or in an emergency; however, the impact of oral disease on overall health and well‐being is concerning (Goode et al., 2018). Oral health disorders are broad and include dental caries, gum disease and other oral cavity problems where risk factors include a poor diet, tobacco smoking and alcohol use (Caton et al., 2016; Fazel et al., 2014; Priyanka et al., 2017; Shekarchizadeh et al., 2013). Oral disease affects the quality of life, disrupting speech, communication, self‐image and social functioning (Mouradian, 2001; Selwitz et al., 2007). Tooth loss and oral pain are common for all age groups experiencing homelessness (Freitas et al., 2019).

A Brazilian study found that poor oral health undermined daily activities amongst adults experiencing homelessness compared to the overall population. Specifically, this is related to difficulty eating, shame and reluctance to smile due to the need for an upper prosthesis (Lawder et al., 2019). Other evidence suggests key barriers to adults experiencing homelessness accessing dental care were structural (such as requirements to register for government dental care and cost of services); organisational (being treated disrespectfully by dental health service providers); and personal (dental care, not a priority, with only emergency care accessed for treatment) (Goode et al., 2018; Mago et al., 2018; Paisi et al., 2019).

Public dental services often have long waiting lists and fixed appointment systems, are costly, and expect clients to adapt to the requirements of the service. Failure to comply, including difficulties clients may face completing appropriate forms or settling outstanding debts incurred for previous treatment, often result in negative responses from staff where the client feels disrespected, judged and blamed (Durey et al., 2016). Unsurprisingly, clients are unlikely to return particularly when other demands such as paying for food compete with oral health needs which subsequently becomes less of a priority (Charnock et al., 2004; Hede et al., 2019). Acknowledging the ‘cultural incompatibility’ of fee‐for‐service models of dental care for low‐income and homeless communities offers an opportunity to consider more appropriate community‐based clinics to better meet the needs of these populations (Wallace & MacEntee, 2012).

One option is to locate dental services near disadvantaged populations with dental professionals being more patient centred in how they deliver services (Christian et al., 2015; Goode et al., 2018). Evidence suggests that meeting this group's urgent oral health needs requires publicly funded preventive and restorative dental care and training dental professionals to work in this context (El‐Yousfi et al., 2019; Freitas et al., 2019). However, progress is slow, often hindered by a lack of political will or complex and unwieldy bureaucratic requirements (Hede et al., 2019; Scrine et al., 2019). Other options include more information about community dental clinics, respect rather than discrimination from the dental and other health providers (Mago et al., 2018), earlier interventions, more focus on prevention and a shared and coordinated approach to the health and well‐being of adults experiencing homelessness (Davies & Wood, 2018). This suggests that, at least in the interim, alternatives are needed to current models of dental care if access to services by this population group is to increase.

Given the plethora of evidence on the need to address social inequalities, Marmot has advocated for more action to improve the health of disadvantaged populations rather than just more research (Marmot, 2015). Inequitable power relations between the socioeconomically privileged and the disadvantaged ‘other’ need disruption from a social justice perspective where each is instead treated as people of equal worth. Translating knowledge about social justice into equitable service delivery can improve access and oral health outcomes for adults who are homeless or at risk of homelessness and contribute to human flourishing (Anderson et al., 2009). This is particularly pertinent when the intersection between poverty, homelessness and suffering can inhibit accessing oral healthcare (Wallace & MacEntee, 2012) yet disadvantaged social groups are often blamed for their poor health, rather than the socioeconomic or structural context in which their lived experience is embedded (Hochlaf et al., 2019).

Dental care in Western Australia comprises various services: private, where treatment may be covered by insurance; public or government‐funded services (including hospital emergency departments where treatment can occur) usually requiring a co‐payment from clients and free dental services provided by Aboriginal Community Controlled Health Services (ACCHS). ACCHS were established in the 1970s to provide culturally appropriate services for Aboriginal Australians. As well as focusing on preventing and treating disease, the services fostered community development and provided educational resources for health professionals (Anderson & Wakerman, 2005; Hunter et al., 2005).

This paper presents findings from a model of oral healthcare using volunteer dentists at a community‐based service, St Pat's that was established to meet the needs of adults experiencing homelessness in Fremantle, Western Australia. Fremantle is a port city within the Perth metropolitan area offering private dental services, one government dental clinic and public and private hospital emergency services.

St Pat's (https://stpats.com.au/) is a not‐for‐profit organisation catering to clients who are homeless, at risk of homelessness, have mental health issues or experience socioeconomic disadvantage. The Day Centre provides access to support services including housing, health, welfare and emergency relief, social activities, education and training. It also offers meals, facilities and specialist services. Service use averages 222 people/weekday and the most common presenting issues are financial difficulties, housing crises, unemployment, mental health and medical issues. Funding for the organisation is received via fundraising activities, donations and government and other non‐government support.

Staff at St Pat's identified a need for dental care in their clients and 2016 established and developed a fully equipped, community‐centred oral health clinic (OHC) funded externally with free clinical care provided by volunteer dental professionals. The OHC is integrated into the existing Day Centre providing a safe, familiar and secure space with flexible access to key services for those most in need. The objectives of this project were to examine whether providing free oral care using volunteer dental professionals and creating a responsive, respectful and trustworthy environment for clients who are homeless or at risk of homelessness is perceived to be effective in meeting their oral health needs (Charnock et al., 2004; Hede et al., 2019). The paper presents findings from a qualitative study investigating service providers and users' responses to setting up, delivering or receiving dental services at this community‐based volunteer OHC.

## 2 METHODS

A social constructivist approach was chosen as it aims to describe reality as constructed by individuals within a specific context. This approach positions the participant at the centre of the meaning‐making process whose voice and interpretations are captured in their interactions with the researcher and are central to examining the topic of interest (Polit & Beck, 2010; Thomas et al., 2014).

Participatory action research guided data collection and analysis which included reflection and action to reduce health inequities and improve health outcomes by involving participants in the process (Baum et al., 2006). Three categories of participants were included: clients (adults experiencing homelessness or at those risk of homelessness) who accessed the dental clinic; service providers involved in the establishment, organisation and/or management of the clinic; and volunteer dental health professionals (Table 1).

**TABLE 1:** Categories of participants.

Category	Participants	Code	Total
Volunteer	Dentists	A	6
	Dental nursing assistant students	E	2
	Other stakeholders	C	5
Clients	Homeless adults	F	18
Service provider	Day Centre staff	B	2
	Oral health clinical reference group (OHCRG)	D	3
	Volunteer centre staff	G	1
	Executive management	H	2
Total			39

Ethics approval to conduct the research was obtained from The University of Western Australia Human Research Ethics Committee (Approval number RA/4/20/4975).

### 2.1 Data collection

Purposive sampling was used to recruit participants from each category via word of mouth, phone or email. Participants were then organised into eight discrete groups (Table 1) to engage in the project. Clients were informed about the project by staff at the Day Centre and researchers followed up those interested inviting them to participate.

Following informed consent, individual semi‐structured interviews lasting 30–60 min were conducted and audio‐recorded at the Day Centre from October 2018 to August 2019. Specific lines of inquiry guided the questions for dental health service providers including how the OHC was established, who was involved, the program's aims, and barriers and enablers to implementation. Other questions related to the OHC's impact on clients and other participants involved in management or service provision. Whilst questions varied between groups, all participants were asked about their perspective and experience of the clinic. Initially, each participant was questioned about the clinic's establishment, but it became clear that clients were not involved and questions for them focused more on the program's impact, enablers and barriers to accessing care, responses to care and oral health outcomes. Interview guides were developed for each group. For example, topics for management related to their experiences setting up the program, perceptions of its impact and whether resources were adequate in terms of ongoing sustainability; for clients topics related to their experience attending the clinic; topics for dental professionals included their motivation to and experience of volunteering in this context. Interviews were transcribed verbatim, de‐identified and imported into NVivo 12 (https://www.qsrinternational.com) to organise and manage data.

### 2.2 Data analysis

Researchers used an inductive approach to analyse raw data specifically related to participants' perceptions and experiences from which themes were derived and classified under two pre‐determined categories ‘establishing the OHC’ and ‘responses to implementation of the clinic’ (Azungah, 2018). Participants' responses were organised into specific groups mentioned earlier (Table 1) to assist in a comparative analysis of key themes emerging within and between groups. Two authors (A.D. and H.L.) independently conducted a line‐by‐line analysis of each interview within each group to identify key themes related to the establishment and impact of the clinic. There was some overlap between groups as several participants belonged to more than one group, e.g., oral health clinical reference group (OHCRG) members also volunteered as dentists. Themes were then compared within and between groups for similarities and differences, discussed and reviewed by all the authors until a consensus was reached.

## 3 RESULTS

Thirty‐nine semi‐structured interviews were conducted across participant groups and responses were organised under the categories of ‘establishing the OHC’ and ‘responses to implementation of the clinic’ (Table 1). Key themes organised under ‘establishing the OHC’ included incremental steps related to funding, creating a welcoming and respectful environment, recruitment and governance and identifying strong principles to underpin patient‐centred care. Key themes under the category ‘responses to implementing the clinic’ included increased client access to oral health services and follow‐up care and positive responses from health providers volunteering at the OHC. Challenges included the OHC's sustainability with uncertainty around funding and recruiting volunteer dentists.

### 3.1 Establishing the OHC for adults experiencing homelessness

#### 3.1.1 Background and aims

St Pat's was instrumental in establishing the OHC with the CEO and chairman of the board, as members of executive management, guiding the process from the beginning. The organisation obtained funding to submit a business case for an OHC for adults experiencing homelessness. Day Centre staff had previously conducted in‐house surveys and interviews identifying clients’ needs and non‐dental health providers involved in other health clinics at the Centre. Findings indicated clients' high oral health needs often require urgent dental care.

**FIGURE 1:**
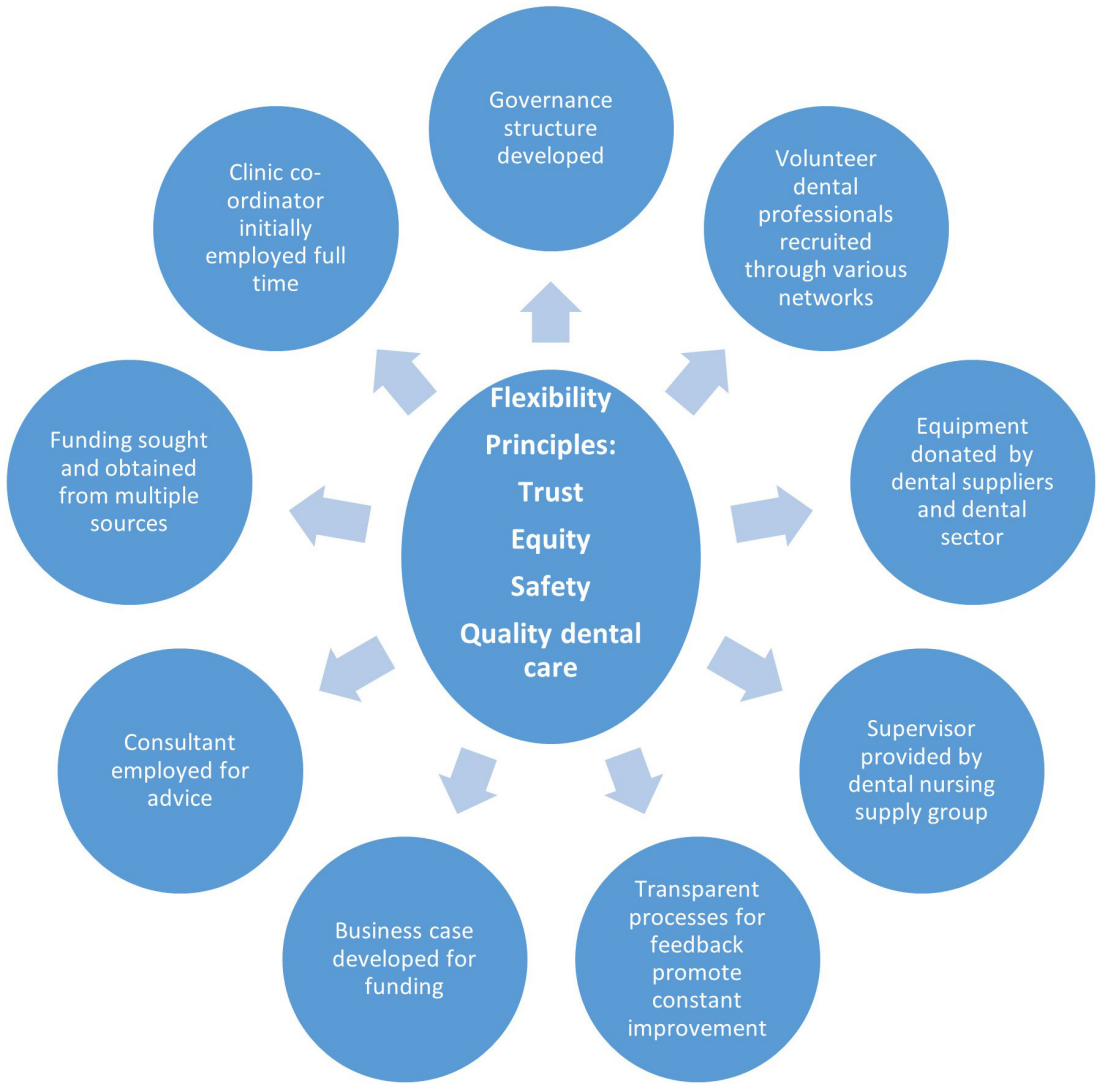
Principles and essential building blocks for establishing and implementing the oral health clinic.

The advisory board's aim in establishing the OHC was to make a meaningful difference in the lives of people who were disadvantaged and marginalised, a group often difficult to access and with complex needs, and to deliver equitable and respectful services. In planning the service, executive management and Day Centre staff emphasised the importance of creating an environment of trust, safety and advocacy to provide quality dental care to clients. Participants from the advisory board involved in designing and setting up the OHC identified a series of steps to build a solid foundation that included systems to appropriately resource services, all guided by a set of strong principles, namely trust, equity, safety and quality dental care (Figure 1). Building blocks included gaining funding for a full‐time OHC coordinator, resources to operationalise the clinic and governance structures.

One health professional stated that the OHC provided a ‘much needed service to a group that otherwise probably wouldn't seek dental services, other than in the emergency situation’. Responses of other OHCRG members and Day Centre staff were keen to ‘dream big’ and set up a fully functioning OHC. This was made possible by engaging with an extensive network of supporters across the dental supplies industry and other funding sectors including government, private, industry and philanthropic sectors who donated or subsidised resources to the OHC. These resources were then used to equip the dental clinic including a dental chair and new OPG (orthopantomogram machine, specialised scanner providing a panoramic view of the jaw and teeth), donated by the dental industry.

Day Centre staff, executive management and OHCRG members considered this high‐quality equipment and rigorous maintenance of the clinic would assist in giving clients the best possible quality service and facilitate a rewarding experience for volunteer dentists. The Day Centre manager had oversight of ensuring compliance with implementing maintenance plans, managing warranties and servicing the equipment, under guidance from the OHCRG.

Members of the executive and Day Centre staff noted the generosity, passion and dedication of designers, donors and volunteers involved in the OHC's implementation.

#### 3.1.2 Creating a welcoming and respectful environment

Creating an environment of trust, safety and advocacy for clients underpinned the delivery of quality dental care. Advisory board participants and Day Centre staff acknowledged the challenges many clients faced complying with public or private services' fixed appointment systems. To offset this, the OHC offered flexible appointment systems including reminders, if possible via mobile phone. As many clients already accessed the Day Centre for other services including meals, social work and recreational activities, offering oral health services within that familiar and trusted setting facilitated access.

#### 3.1.3 Governance structure

The governance structure of the OHC involves the executive, the OHCRG, management and administration and a community reference group that forms the committee. The committee's roles included reviewing data, procedures, risk management and responding to feedback. Quarterly meetings involve the CEO, clinical lead, Day Centre manager and clinic coordinator, and various other stakeholders from the government, the tertiary sector, the dental profession and the services industry. The OHCRG ensured clinical governance and quality of dental care are maintained in the clinic, meeting bi‐annually to discuss clinical practice and other issues to ensure its smooth running.

Clients and volunteers were encouraged to provide feedback and formal complaints and grievance procedure were initiated to ensure transparency. For example, a complaint about a dentist would be recorded, appropriate documentation completed and sent through to management and, if necessary, to the Board for a response.

A strategy to facilitate good governance required employing an OHC coordinator.

You need to have that key person in the mix that can be the liaison between everyone, because if you took a coordinator out of the mix and you were only dealing with volunteers, volunteer reception in the health clinic, volunteers coming in, volunteer people in, you don't get the connecting thread. (Day Centre Staff).

The OHC coordinator's role involved scheduling clinic days and client appointments, managing the database, maintaining client records and service agreements for volunteer dental professionals, managing procedures around recruiting volunteers including induction and delivering services, supervising volunteer receptionists, and, crucially, liaising between volunteer dentists and clients. Whilst dentists rarely missed their clinics, they were cancelled if dentists were unable to attend. Long‐term funding for the coordinator role is currently not secured.

#### 3.1.4 Recruitment

A stakeholder involved in recruiting volunteer dentists noted that existing services within the community were not meeting the needs of people who were:

…homeless, disadvantaged economically and socially, … A lot of them can't even afford subsidised treatment that they could receive at government dental clinics (Other stakeholder).

Dental professionals were recruited as volunteers via word of mouth, advertising in dental newsletters, promoting the OHC at conferences or in articles in dental industry newsletters and via peak bodies such as the Australian Dental Health Foundation, a leading recruiter of dentists. The duration of individual volunteering at the OHC varied from several months to 2 years. A local training centre provides and supervises student dental assistants. Clients are recruited via word of mouth or cross‐referrals from other clinics at the Day Centre.

#### 3.1.5 Challenges

Participants were concerned about time‐limited funding and recruiting enough volunteer dentists to ensure the ongoing viability of the OHC. Whilst dental assistants provided services, some dentists found student nurse involvement and not having a regular dental nurse impacted their productivity. However, student nurses appreciated working with this client group.

It did open my eyes a little to see how people live and how they are and what they do not have, what we have, and stuff like that. (Student Dental Nurse).

### 3.2 Responses to the OHC

Participants from executive management and Day Centre staff felt the OHC has met its goals to deliver services adapted to the needs of clients experiencing or at risk of homelessness, rather than the more usual ‘one size fits all’ approach.

The essential factor to my mind is that it is in that trusted environment (Executive Management).

Clinic data included treatment for dental caries, pain, gum disease and edentulism via the use of fillings, plaque removal, professional teeth cleaning, extractions including wisdom teeth, root canal treatment and provision of partial and complete dentures. Volunteer dentists indicated they chose functional treatments to ensure clients were pain‐free.

You want the filling and the work to last and not to cause pain, and there are time constraints and knowing that the patient may or may not be able to or want to come back again. So it is trying to kind of get as much done in the appointment to a high standard that you know is going to be functional. (Volunteer Dentist).

Whilst dentists often carried out more extractions than in private practice, clients were offered and fitted for dentures at little or no financial cost.

#### 3.2.1 Accessing the OHC

Most clients identified free dental care as a key factor in accessing the clinic. Some were in debt or could not afford dental care after they had paid essential expenses. Other health and social services located at the Day Centre facilitated referral pathways to the OHC for clients needing dental care. Clients appreciated being able to access the OHC at the Day Centre and complete treatment. Clients rated the clinic as very good to excellent on a scale of 1–10 with 10 being excellent. One client rated the clinic ‘7 or 8’ the rest scored 8 or higher with 6 rating it at 10. Clients stated they had become more informed about oral health and, of the 18 interviewed, all had attended follow‐up appointments.

Because of all the plaque I also had gum disease because of it all, so they had to clean it all and now my gums are all good. Everything is all good, and I'm trying to keep all of that up. (Client).

Whilst free dental care was significant in the rating, other factors were also noted including the caring and welcoming atmosphere, professionalism and lack of judgement from staff. In some interviews, the judgmental attitude experienced at other dental services was contrasted with clients feeling respected and included in decisions about their treatment at the OHC.

They ask what I would prefer. They ask, ‘Is there anything wrong?’ They check out the situation in all aspects … So it is good. It is a friendly interaction rather than, ‘I am dominating you. You have got to do as I say. (Client).

Clients appreciated the flexible appointment system, relatively short waiting times, text reminders and phone calls and the stand‐by system for appointments if there were cancellations, useful for those without mobile phones. Clients seemed to accept that those needing emergency dental care were prioritised. Morning reminder calls meant staff became aware of ‘no‐shows’ early enough to offer appointments to other clients on ‘stand‐by’ or who were waiting in the queue at the day centre ensuring maximum use of dentists' time. Several clients said they would come for check‐ups rather than wait till they had a problem.

#### 3.2.2 Oral health outcomes

Most clients commented on the benefits they received from dental care that were transformative as well as practical such as being able to chew food:

It will be awesome to be able to smile with some teeth, yeah. It is a big bonus. (Client).

Since they took my teeth out I have put on weight. I have gotten healthier, yeah, because I could not eat with them wiggly like that because the infection would set in underneath the gums in between the teeth. (Client).

Others felt confident enough to apply for a job following treatment at the OHC.

I wanted to go back to work because I'm a teacher's assistant and you cannot get a job when your teeth are all like that. No one wants to hire you in a primary school when you look like that, yeah. … I'll get another job hopefully, yes. (Client).

Improvements were often most dramatic following the application of dentures, either because the existing teeth were in poor condition or because the client was edentulous, some for many years.

An intention when setting up the clinic was to shift focus from managing clients' emergency dental needs to engaging them in ongoing restorative treatment, education and changed behaviour to prevent further problems seemed to be working.

I've sort of been looking after my teeth, brushing, where before just once in a while or before you go to the doctor you would brush your teeth, but now I've been morning and night. (Client).

The transformative effect on clients of improving their oral health was noted by volunteer dentists:

I have seen a vast majority of my patients go on to find stable housing and even gainful employment after having their teeth rehabilitated. Their own personal esteem and confidence is palpable as the treatment progresses and I would say the impact of our work can be life changing. (Volunteer Dental Professional).

Just this guy, he said that he didn't have any job… he said that he was eagerly waiting for his dentures because he has an interview so that he can get a job, because it was his front tooth, like, front two teeth. When we gave him the denture he was like over the moon. (Volunteer Dental Professional).

Helping to improve oral health for those who were homeless also reaped rewards for those who volunteered at the OHC:

They are absolutely so emotionally and overwhelmingly grateful. So, yeah, it is just a wonderful thing to be involved in. (Other Stakeholder).

One of the ladies there [client experiencing homelessness] gave us like a thank you card for making her smile, you know. It was so good. (Other Stakeholder).

The opportunity to give back to their community and make a difference by improving the oral health of adults experiencing homelessness was a reward in itself with volunteers prepared to recommend the experience to others:

The real positive message about the whole thing is that you can have such a more profound impact than we are used to seeing in normal day‐to‐day drill‐and‐fill dentistry. You can really change the course of people's lives when they feel like they've been invested into, in their appearance. (Volunteer Dental Professional).

#### 3.2.3 Ongoing challenges

Despite the huge demand for oral healthcare in this target population and the positive response to the dental clinic across groups, the sustainability of the OHC is not guaranteed. Ongoing funding for currently employed staff is not assured, and neither is any back‐up if one staff member is either sick or on leave. When that happens, no dental clinics are booked in. Other challenges are structural and relate to the OHC's ability to meet Commonwealth Government and other funding model requirements for oral care in future.

At an organisational level, several groups considered employing a permanent dental assistant was necessary to ensure continuity of patient care, the efficient organisation and management of the dental clinic and the capacity to supervise dental assistant students. Whilst some participants suggested recruiting more volunteer dentists, space at the day centre is limited with no room currently for expansion. Some dentists would like greater throughput of clients but were constrained by various factors including clients who failed to attend appointments.

When asked whether a model of care that specifically meets the needs of this target population might relieve the government from the responsibility to provide dental services, one participant responded:

I think there is a need for both … We'll try and gently encourage [clients] to go to the government dentist if we think that that is more appropriate for them. Certainly the need for dentistry is so huge that we couldn't say, ‘Well, we are going to be the stand‐alone’. … So I think there is a need for both, and it would be nice to see this kind of model maybe even being replicated [elsewhere]. (Day Centre staff).

## 4 DISCUSSION

A major finding across all groups was the positive response to establishing and implementing the OHC in providing dental care to adults experiencing homelessness. Our findings provide evidence that using volunteer dental professionals at a community‐based OHC can fill a gap in care by meeting the needs of adults experiencing or at risk of homelessness who may be reluctant or unable to attend other dental services. Clients accessing this community‐based clinic benefited from the treatment, information about prevention and follow‐up care that in many cases transformed their lives. Clients commented on being treated respectfully and with care, reflecting some of the core principles underpinning the OHC including equity, trust, safety, flexibility and offering high‐quality dental care, including providing dentures to those in need. However, uncertainty around future funding for the OHC and the ongoing recruitment of volunteer dentists were a concern.

However, evidence suggests that system‐level barriers such as the cost of care for clients, and disincentives for dentists to work in this area due to poor remuneration rates, prohibit many adults experiencing homelessness from accessing public dental clinics. Instead, rather than seek comprehensive dental care (Coles & Freeman, 2016), many access hospital emergency departments for non‐traumatic dental problems (Cohen et al., 1996; Figueiredo et al., 2016).

Given the numerous challenges facing adults experiencing homelessness, our findings support those from a systematic review on strategies to improve oral health in this population group, particularly around flexible appointment systems (Goode et al., 2018). Flexible appointment systems were found to be more effective in clients returning for follow‐up care in a dedicated oral health service located in a multidisciplinary health centre rather than a mobile clinic (Simons, 2003). By responding to their immediate needs and being sensitive to the socioeconomic and psychosocial factors disrupting the lives of adults experiencing homelessness (Caton et al., 2016), our findings supported those of Van Hout and Hearne (2014) who identified that enablers to dental visits included knowing your dentist and having a dentist onsite in healthcare settings.

Our study supports other evidence that volunteer dental professionals working with disadvantaged social groups appreciate the opportunity to give back to the community (Wallace & MacEntee, 2013). Patel et al.'s (2015) research into dentists volunteering to work in remote Aboriginal communities also reflected this finding by providing dental services where previously they were non‐existent. Whilst our findings indicate that the St Pat's OHC clearly meets a need, responses from management suggested the model was not sustainable with recruitment and funding issues an ongoing concern despite this population group often reluctant to access public dental services. This raises questions about the need for appropriate strategies at the level of policy and practice to increase dental workforce capacity and provide equitable services to a disadvantaged group with complex needs (Mouradian, 2006).

Whilst volunteer models can fill a gap that other services do not reach, at least in the interim, they also set a standard for services that are community centred, equitable and respectful. We suggest that our findings offer principles and key building blocks to creating a safe and trusted environment to increase access and deliver high‐quality oral healthcare to adults experiencing or at risk of homelessness.

### 4.1 Limitations

Whilst participation in this project was voluntary, we acknowledge that purposive sampling may not have captured patients or volunteers dissatisfied with the dental services offered by the clinic. We also acknowledge the risk of potential response bias of those attending the clinic or providing services for the clinic to support its aims. This risk was mitigated by having direct contact with participants, building rapport and trust, controlling the ‘emotional tone’ of questions and debriefing and discussing response bias with the research team (Bergen & Labonte, 2020; Fisher & Tellis, 1998). There may have been other limitations. For example, the clinic was located in a successful day centre but the dynamics of the overall centre were not explored in detail. In terms of analysis—having pre‐determined questions may be a limitation but we did find it helpful for this context.

### 4.2 Conclusion

The sustainability of volunteer models of dental care is not assured given the unpredictability of funding to secure key service providers and uncertainty around the ongoing recruitment of volunteers. However, our evidence suggests that creating an environment of respect, trust and safety for clients who are homeless or at risk of homelessness is an important step in increasing the likelihood they will access oral healthcare, return for follow‐up treatment, factors that can subsequently lead to an improved sense of health and well‐being.

## AUTHOR CONTRIBUTIONS

All authors conceived the study contributed to the proposal, discussed the analysis, and contributed to the paper; Angela Durey led the analysis and drafted the first version of the paper.

## CONFLICT OF INTEREST

The authors have no conflict of interest.

## FUNDING INFORMATION

Joint funding from St Patrick's Community Support Centre and the Sisters of St John of God.

## ETHICS APPROVAL

Ethics approval to conduct the research was obtained from The University of Western Australia Human Research Ethics Committee (Approval number RA/4/20/4975).

## Data Availability

Research data are not shared (for ethical reasons).
